# Interoception as a Mechanism Linking Anxiety, Cardiovascular Disease, and their Co-Occurrence

**DOI:** 10.1007/s11886-026-02421-0

**Published:** 2026-09-24

**Authors:** Brittany L. Keller, Samantha G. Farris

**Affiliations:** Department of Psychology, University of New Jersey, 1 Spring Street, Suite 200, New Brunswick, NJ 08901 USA

**Keywords:** Interoceptive accuracy, Interoceptive attention, Cardiovascular disease, Anxiety, Hypervigilance

## Abstract

**Purpose of Review:**

This review summarizes current research on interoception in cardiovascular disease (CVD), highlights the clinical implications of interoceptive processes in CVD management, and provides recommendations for prescribing symptom monitoring based on findings.

**Recent Findings:**

Compared to healthy controls, individuals with CVD demonstrate low actual interoceptive accuracy and high perceived interoceptive attention, though more rigorous methodology is needed. This interoceptive profile, particularly high interoceptive attention, has also been recently found to be predictive of health anxiety.

**Summary:**

Though interoception research has primarily utilized cardiac-based measurements, there is a dearth of research examining interoception in individuals with CVD. Research that has been conducted in this population has found that individuals with CVD show interoceptive profiles that might increase risk of health anxiety, though more work is needed. We recommend that providers give specific instructions for symptom monitoring (e.g., duration, method, signs to seek medical attention) to minimize uncertainty during CVD management.

## Introduction

Cardiovascular disease (CVD) and anxiety are commonly comorbid – about 16% of individuals with CVD meet criteria for at least one anxiety disorder, and individuals with CVD meet criteria for generalized anxiety disorder and panic disorder at nearly two and a half times the rate of the general population [1]. The co-occurrence of these conditions has important clinical implications, as anxiety has been associated with poorer cardiac-related outcomes for individuals with CVD. For example, anxiety disorders are associated with increased risk of recurrent cardiac events [2] and a 26% increase in risk for cardiac mortality in individuals with CVD [3]. Additionally, in individuals with CVD, the presence of anxiety disorders (compared to none) is associated with more severe CVD symptoms and poor health behaviors which increase secondary cardiovascular risk, including lower engagement in moderate-to-vigorous physical activity [4], lower medication adherence [5], and more disrupted sleep [6].

Interoception, or the processes used by the nervous system to sense and make sense of internal stimuli [7], is a mechanism implicated in both CVD and anxiety [8, 9]. Interoception is necessary for regulation of internal state via homeostasis and allostasis, processes which are crucial for the maintenance of physical and mental health [7]. Disruptions in interoceptive processes are thought to contribute to physical and mental health conditions by preventing the body from properly adapting following environmental change [8, 10]. As a result, disruptions in interoception contribute to physiological changes in the cardiovascular system that cause physical symptoms (e.g., shortness of breath, heart palpitations, chest pain [8]) that are present in both CVD and anxiety. Additionally, interoceptive processing is crucial for detecting physical symptoms and doing so accurately, and is therefore theoretically relevant to both disease management and health-related anxiety. For example, impairments in interoceptive processing can alter one’s ability to accurately detect symptoms of physical disease and/or recognize physical sensations as disease-relevant symptoms [8]. Low accuracy and/or attention to sensations could have a negative impact on the timing and course of treatment, as detecting physical symptoms and their intensity is crucial for informing diagnoses, determining the timing of interventions, and adjusting medications for CVD [11, 12]. In contrast, individuals who exhibit extremely high levels of attention to body sensations could become hypervigilant to small in magnitude or benign changes in body state (i.e., not related to a medical condition) [13]. On the one hand, vigilance to bodily states is adaptive and evolutionarily advantageous, as awareness to the body helps to identify physical symptoms that might represent a threat to survival. However, excessive vigilance (i.e., hypervigilance) to body sensations is maladaptive and shown to be linked with health-related anxiety [14], including cardiac anxiety [15, 16], and therefore might also be maladaptive for CVD management.

Understanding whether (and if so, how) interoceptive processes are altered in the context of CVD could provide useful insight into approaches for improving disease management that does not reinforce anxiety. Therefore, in the current review we (1) provide a brief overview of the current conceptualizations of interoception and methods for studying it; (2) summarize the available literature examining interoception and anxiety; (3) summarize all available research on interoception in the context of CVD; and (4) consider how CVD management strategies might be modified to account for the role of interoceptive processes in CVD and anxiety.

## What is Interoception?

Interoception (also known as *interoceptive awareness*) is a broad construct that refers to the processing of internal stimuli (i.e., interoceptive signals) by the nervous system [7]. Interoceptive signals arise from all body systems (e.g., cardiovascular, respiratory, muscular, endocrine) and are relayed to the brain via complex neural pathways to support homeostatic and allostatic regulation [17]. Broadly, interoceptive signals include those that can be consciously perceived (e.g., muscular effort, cardiac sensations, respiratory strain) as well as those which operate outside conscious awareness (e.g., blood pH; [18]). Interoceptive signals that can be consciously perceived manifest as physical signs and symptoms related to internal state (e.g., racing heart, dizziness, pain).

The brain senses and integrates interoceptive signals to create a representation of current body state using specific processes (also sometimes described as “domains” [7]) which fall under the broader umbrella of interoception. These interoceptive domains are thought to describe the processes used to sense and integrate all interoceptive signals, though it is worth noting that it remains unclear whether the neural pathways used for these processes are overlapping or distinct across unrelated (e.g., gastric and cardiac) and related (e.g., cardiac and respiratory) body systems (see the review by Khalsa et al. [7]). Conceptualizations of specific features of interoception are inconsistent across the literature [18, 19]. The most used models of interoception [7, 19, 20] vary in how many and which features are considered core processes of interoception (see Table 1 for a description of the core processes of interoception across each of the major models). This heterogeneity in models of interoception complicates synthesis of findings from extant research as well as approaches for further investigations of interoceptive processing across body systems, and thus highlights the need for a centralized model of core interoceptive processes.

**Table 1:** Interoceptive domains and methodology

Interoceptive Domain^1^	Definition	Garfinkel et al. [20]	Khalsa et al. [7]	Murphy et al. [19]	Example methodology^2^
Accuracy (objective accuracy)	ability to correctly perceive interoceptive stimuli	X	X	X	Modified HCT^b, 3^ [21–23]; modified HDT^b, 3, *^[23, 24]
Sensibility (subjective accuracy)	perception of one’s interoceptive accuracy	X	X	X	BPQ^s, 3,*^[25]; Confidence ratings^s^ [20]
Insight	agreement between actual and self-reported accuracy	X	X		ROC curve analysis^b^ [26]
Attention (objective)	tendency to focus on internal sensations		X	X	Experience sampling methods^b, 3^ [19]; EEG (HEP modulation [27–29]) ^b^
Attention (subjective)	perception of one’s interoceptive attention			X	BPQ^s, 3, *^ [25] IATS^s, 3^ [30]; BVS^s, 3^ [31]
Detection	ability to determine the presence or absence of a sensation		X		HDT^b, 3, *^; Inspiratory occlusion and resistive load paradigms^b*^ [32]
Magnitude	perception of intensity of a sensation		X		Bolus isoproterenol infusions^b*^ [33]; Inspiratory occlusion and resistive load paradigms^b*^
Discrimination	ability to tell the difference between sensations		X		Bolus isoproterenol infusions^b*^; BVS^s, 3^ [31]

Many of the interoceptive domains (e.g., accuracy, detection, magnitude) proposed by the Khalsa model are not mutually exclusive. See “What is interoception?” section for a discussion of issues with this model

^1^ “Self-report scales” (proposed as a domain in the Khalsa model) exclusively reflect a measurement method rather than a distinct aspect of interoception and therefore have been omitted from this Table ^2^ These examples were selected because we have determined that they have the best construct validity. ^3^ See “Measuring Interoception” section for information about these measures and their limitations. ^b^ behavioral/objective method; ^s^ subjective method; ^*^method has been used to measure multiple domains

HDT = Heartbeat Detection Task; HCT = Heartbeat Counting Task; BPQ = Body Perception Questionnaire; IATS = Interoceptive Attention Questionnaire; ROC = receiver operating characteristic; BVS = Body Vigilance Scale; EEG = electroencephalography; HEP = heartbeat evoked potential

One of the more prominent models of interoception was proposed by Khalsa et al. [7]. This model was created to provide a comprehensive framework of interoceptive processes while consolidating variable nomenclature and definitions across the literature. There are 8 proposed domains in this model (see Table 1), with the most studied aspects being interoceptive accuracy and sensibility. Although this model provides a comprehensive overview of interoceptive processes at a neurological level and helps standardize construct definitions and terminology across certain literatures, there are significant issues with construct validity and associated measurement issues. For example, there are interdependencies in some of the proposed constructs, which muddies both conceptual and measurement discrimination. For instance, an interoceptive signal needs to be detected to determine its magnitude, whether it is distinct from another interoceptive signal, and to accurately perceive the signal. Additionally, “self-report scales” is an interoceptive domain according to this model, though this is not an interoceptive process itself but rather a methodological approach to measuring interoceptive constructs.

Therefore, we recommend the 2 × 2 factorial model of interoception proposed by Murphy et al. [19] given its conceptual and measurement clarity, as well as clinical utility. According to this model, there are two core interoceptive processes – accuracy and attention – and these constructs can be further understood by considering one’s perceived and actual ability. From a research perspective, this conceptualization offers a more parsimonious perspective and strong construct validity. The Murphy model also offers direct clinical application, as it provides insight into the relevance of actual versus perceived interoceptive abilities, which we describe later in this review (see “Clinical Implications and Recommendations” section). For these reasons, we discuss interoceptive processes – with a particular emphasis on cardiac interoception given its relevance to CVD – through the lens of the 2 × 2 factorial model in the current review.

### Measuring Interoception

To measure the interoceptive domains proposed by the 2 × 2 factorial model of interoception, objective and subjective methods for assessing accuracy and attention have been developed. Although an in-depth description of methods and their limitations are beyond the scope of this paper, we provide a brief overview of common methods for assessing each of the four core interoceptive processes, with considerations about their use. Actual (objective) interoceptive accuracy is the most commonly measured domain of interoception, with methods often leveraging interoception in cardiac domains [34]. Popular methods for assessment determine actual interoceptive accuracy based on the ability to correctly detect whether a heartbeat occurred and, if so, when it occurred. There are two common methods for assessing objective interoceptive accuracy. The heartbeat counting task (HCT, also called the Schandry task; [21]) involves instructing an individual to count the number of heartbeats they detect during a specific time period while heartrate is being recorded. Accuracy score is derived by comparing the number of counted versus recorded heartbeats. Because of various biases in this task [34, 35], a proposed modification involves requiring individuals to tap a key when a heartbeat is detected rather than counting [24, 25], which allows for evaluation of accuracy in detecting the presence and rhythm of heartbeats. In the heartbeat detection task (HDT; [26]), an individual is instructed to adjust an externally-generated repeating stimuli (e.g., metronome) to match their own heart rhythm while heartrate is recorded. Accuracy is determined by how closely an individual can align the external signal with their recorded heart rhythm. Perceived interoceptive accuracy is typically assessed by asking an individual to rate their confidence in their accuracy on the HCT or HDT [19, 20]. Because the HDT involves external and internal perceptual processing, the modified HCT is more commonly used as it requires only internal processing. Indeed, modified HCT is likely the strongest method for measuring interoceptive accuracy to date, as it allows for determining accuracy in number of beats counted in addition to *when* the beats were detected and can therefore provide insight into accuracy in perceiving heartbeats and changes in their rhythm [34].

In contrast to the more established study of objective measures of interoceptive accuracy, few methods have been developed to study objective interoceptive attention. Recent research within the neuroscience field has suggested that attention to heartbeats results in modulated heartbeat-evoked potentials (HEPs; [27–29]). Approaches for measurement of HEPs varies across the literature [36] but typically involves simultaneous collection of data from electroencephalography with electrodes placed on the scalp at sites CZ, F3, FZ, and PZ and from electrocardiogram (ECG; [37]). The epochs for HEPs are measured in reference to each R peak of the ECG (which represents each heartbeat) and are typically set to occur 200ms before to 600ms after the R peak [36]. Another potential objective method for assessing interoceptive attention is ecological momentary assessment (EMA) [19]. While EMA approaches utilize self-report methods, they are understood to reduce response bias by asking participants about current or recent past (e.g., past 5 min) experiences [38]. In the context of interoceptive attention research, EEG appears to be the current strongest method for assessing objective interoceptive attention during in-lab paradigms, while EMA methods are preferable for collecting naturalistic data about interoceptive and exteroceptive attention.

Perceived (subjective) interoceptive attention has been assessed using a wide variety of self-report measures (see Table 1). Although all self-report measures are understood to be limited by response bias, measures used for assessing perceived interoceptive attention have other significant limitations which must be considered. For example, one of the most used methods for assessing perceived interoceptive attention is the Multidimensional Assessment of Interoceptive Awareness-2 (MAIA-2 [39]). The MAIA-2 contains items related to noticing and shifting attention away from sensations but also contains many items that assess responding emotionally to sensations, using interoceptive attention to cope with distress, and trusting the body. Therefore, the MAIA-2 taps not only interoceptive attention, but also appraisal of and emotions related to sensations. Thus, there is a concern about construct overlap/contamination and limited discriminant validity. Even the items that do assess interoceptive attention also tend to query about “comfortable” and “uncomfortable” sensations, which introduces valence and inherent judgment (cognitive processing) of interoceptive signals rather than true attentional deployment. The Cardiac Anxiety Questionnaire (CAQ) is another commonly used measure (particularly in anxiety research) which has been used to capture heart-focused attention via the attention subscale [16]. While the CAQ is useful in highlighting the relevance of attentional processes to cardiac anxiety, the attention subscale includes behavioral items (i.e., checking heartrate) that muddy content validity when used as a measure for assessing attention. Other self-report measures are also limited in that they capture only specific aspects of interoceptive attention (e.g., attention to specific sensations [25, 30] or attention during specific situations [25, 39]) or contain items that assess other interoceptive processes (e.g., detection) in addition to interoceptive attention [31]. Therefore, subjective measurement of interoceptive attention must be carefully considered if using existing tools and future measurement development work could meaningfully address these limitations.

## Anxiety and Interoception

Anxiety disorders are a class of mental health disorders characterized by excessive fear or worry and often include the presence of somatic symptoms (e.g., racing heart, shortness of breath, lightheadedness [40]). Disruptions in interoception are understood to play a role in anxiety disorders [7, 17]. For example, compared to individuals without anxiety, individuals with anxiety report experiencing somatic sensations more often and more intensely, as well as engaging in more negative appraisal of somatic sensations [25, 41]. It has been theorized that increased reporting of somatic sensations in anxiety disorders is influenced by disruptions in interoceptive accuracy and attention. Garfinkel et al. [42] theorized that individuals with anxiety show decreased interoceptive accuracy coupled with heightened attention to body sensations. The theoretical relevance of interoception in anxiety disorders has led to a large body of empirical research on interoceptive processes – particularly actual accuracy and perceived attention – and their association with anxiety disorders [43–45]. Given that there have been several recent reviews of literature examining interoception and accuracy, we provide a brief summary of the state of this literature here.

There is a large body of extant research examining interoceptive accuracy in the context of anxiety, with overall findings contrasting with prominent theories of the role of interoceptive accuracy in anxiety. Two recent reviews and meta-analyses examining actual interoceptive accuracy in the context of anxiety have found no consistent association with trait anxiety [43, 45] and state anxiety [43] in both healthy controls and clinical samples across the literature (for a more in-depth discussion about these results, see Adams et al. [43] and Desmedt et al. [45]). Additionally, no consistent association emerged when accounting for potential moderators such as the presence of diagnosed anxiety disorders, severity of anxiety symptoms, or method used to assess actual interoceptive accuracy (i.e., HCT or HDT [43]). Interestingly, recent research has found that anxious individuals, but not healthy controls, showed differences in ability to accurately detect interoceptive and exteroceptive signals [46]. Thus, anxiety might be associated with relative within-person increases in interoceptive accuracy compared to exteroceptive accuracy, though this line of work is still in its nascency.

Perceived interoceptive attention has been a particular focus of research investigating the role of interoception in anxiety, with more consistent findings than that on interoceptive accuracy. Recent meta-analyses have found that frequency of attending to body sensations and difficulty shifting attention away from body sensations are positively associated with anxiety [44]. Additionally, heightened attention to body sensations has been associated with increased anxiety sensitivity and severity of anxiety symptoms in both clinical and non-clinical samples [31, 44, 47]. Interoceptive attention has also been positively associated with health anxiety [14, 48] and cardiac anxiety [15]. It should be noted that work by Trevisan et al. [48] suggests that anxiety is positively associated with “maladaptive” interoceptive attention (e.g., hypervigilance to body signals that might indicate illness) and negatively associated with “adaptive” interoceptive attention (e.g., trusting the body, listening to the body for guidance). However, the distinction between “maladaptive” and “adaptive” attention is unclear, as the “adaptive” processes described by Trevisan and colleagues reflect *appraisal* of body sensations rather than attentional processes themselves (as described in the “Measuring Interoception” section). Additional research is therefore needed to understand when and how heightened perceived interoceptive attention itself, which evidence has shown characterizes anxiety, is helpful versus harmful.

## Cardiovascular Disease and Interoception

Despite the theoretical relevance of interoception in CVD and the prominence of methods that utilize cardiac domains to assess interoception in healthy individuals, there has been limited work done to assess interoception in the context of CVD. Below, we review recent research that has examined interoceptive accuracy and attention in the context of CVD. Table 2 provides a description of relevant research, including populations studied, interoceptive domains assessed, methodology, and findings.

**Table 2:** Literature on interoception and cardiovascular disease

Author [reference]	Population(s)	Interoceptive domains measured				Method	Findings
		Actual accuracy	Perceived accuracy	Actual attention	Perceived attention		
Owens et al. [49]	Adults w/ PoTS Adults w/ VVS Healthy controls	X	X			HCT, confidence ratings	Actual accuracy for adults w/ PoTS or VVS > healthy controls. No significant differences for perceived accuracy
Abrevaya et al.[50]	Adults w/ HHD Adults w/neurological conditions Healthy controls	X	X	X		Modified HCT, confidence ratings	All interoceptive domains measured distinguish adults w/ HHD from adults w/neurological conditions and controls
Yoris et al. [51]	Adults w/ HHD Healthy controls	X				Modified HCT	Actual accuracy for adults w/ HHD < healthy controls
Miyazaki et al.[52]	Adults w/ CVD	X				HCT	Moderate-to-high levels of actual accuracy observed
Hoffman et al. [53]	Adults w/ CHF Healthy controls	X	X		X	HCT, confidence ratings*, MAIA	No significant differences in any domains
Limonova et al. [54]	Adults w/ symptomatic and asymptomatic PVCs	X		X		HCT, Modified HCT	Actual accuracy for symptomatic individuals > asymptomatic individuals, only when measured by HCT (not modified HCT). Heightened actual interoceptive attention is associated with higher symptom severity
Locatelli et al. [55]	Adults w/ CVD				X	MAIA-2	Moderate levels for “noticing” and “attention regulation” subscales. Low levels for “distracting” subscale
Varela et al. [56]	Adults w/ ICC Healthy controls				X	MAIA-2	Adults w /ICCs > normative controls for “noticing” and “attention regulation” subscales. Adults w/ ICCs < normative controls for “distracting” subscale
Yu et al. [57]	Adults w/ CHF				X	MAIA-2 C	Moderate levels for “distracting” subscale. Moderate-to-high levels for “attention regulation” subscale

CCP = cardiac chest pain; NCCP = non-cardiac chest pain; PoTS = postural orthostatic tachycardia syndrome; VVS = vasovagal syncope; HHD = hypertensive heart disease; CVD = cardiovascular disease; CHF = coronary heart failure; PVC = premature ventricular contraction; ICC = inherited cardiovascular conditions; HCT = heartbeat counting task, HDT = heartbeat detection task. ^*^ method has been used to measure multiple domains

### Interoceptive Accuracy

Measurement of actual interoceptive accuracy has been a major focus of research on interoception in individuals with CVD. Recently, there has been some research to suggest that individuals with CVD and with symptomatic premature ventricular contractions (a common cause of heart palpitations; PVCs) demonstrate moderate-to-high accuracy when performing the classic HCT [52, 54]. Additional work has suggested that actual interoceptive accuracy distinguishes individuals with hypertensive heart disease from healthy controls and individuals with other physical health conditions [50]. It should be noted that evidence for increased actual interoceptive accuracy in people with CVD is mixed across the literature, which might be a result of heterogeneity in methods for assessing actual interoceptive accuracy as well as types of CVD populations being examined. There have been a couple of studies which found that individuals with CVD (e.g., postural orthostatic tachycardia syndrome, vasovagal syncope [49], hypertensive heart disease [51]) show lower actual interoceptive accuracy than healthy controls using HCT and modified HCT paradigms. In contrast, one study which used the HCT has reported no significant difference between individuals with chronic heart failure and healthy controls [53], though given its methodological limitations, evidence from the classic HCT paradigm should be interpreted with caution. In summary, research utilizing more robust measurement methods suggests that there are meaningful differences in actual interoceptive accuracy between individuals with CVD and healthy controls, which underscores the need for future research to examine clinical implications and disease-specific factors that might impair accuracy for individuals with CVD.

Considerably fewer studies have examined perceived interoceptive accuracy in the context of CVD. Two studies [49, 53] have found that individuals with CVD show no significant differences in perceived interoceptive accuracy when compared to healthy controls, with participants in both groups reporting moderate confidence in their performance on the classic HCT. In contrast, one study that utilized a modified HCT [50] found that perceived interoceptive accuracy was a key domain for distinguishing individuals with hypertensive heart disease from healthy controls and individuals with neurological conditions. Preliminary examinations of perceived interoceptive accuracy that leverage more robust methods of assessment suggest meaningful differences between individuals with CVD and healthy controls. This area of investigation is still nascent and would benefit from additional work aimed at characterizing perceived interoceptive accuracy in individuals with CVD and examining how perceived interoceptive accuracy in this population compares to that of controls.

### Interoceptive Attention

There have been very few studies to date that examine interoceptive attention in the context of CVD, and all have done so by examining modulated HEPs during HCT and/or HDT tasks. Limonova et al. [54] found that lower changes in HEP modulation during interoceptive accuracy tasks compared to rest – which indicates greater sustained attention to heartbeat at rest - were found to predict higher symptom severity for individuals with PVCs. Other work utilizing EEG data has indicated that actual interoceptive accuracy is a key factor that distinguishes individuals with CVD from healthy controls and individuals with neurological disorders [50]. Therefore, EEG data provides preliminary evidence of the relevance of actual interoceptive attention in CVD. More work is needed, both via in-lab and naturalistic paradigms, to better understand how individuals with CVD attend to interoceptive signals (e.g., frequency of attending to signals, duration of focus), and whether this is different from actual interoceptive attention observed in healthy controls.

Current research examining perceived interoceptive attention in individuals with CVD has exclusively used either the MAIA [58] or the MAIA-2 [39], and therefore reflects general interoceptive attention. Given the considerable limitations of these measures (see “Measuring Interoceptive Attention” section), here we highlight subscales that only contain items that assess attentional processes. Recent research has found that individuals with CVD report moderate-to-high perceived ability to notice sensations [55], coupled with a low-to-moderate ability to shift attention away from sensations that are deemed uncomfortable [55, 57]. Consistent with the attentional styles suggested by these studies, Varela et al. [56] found that individuals with inherited cardiac conditions reported higher perceived ability to notice sensations and lower perceived ability to distract from sensations than healthy controls. In contrast to the findings by Varela et al., Hoffman et al. [53] found no significant differences in perceived interoceptive attention between individuals with congestive heart failure (CHF) and healthy controls. Therefore, preliminary evidence suggests that individuals with CVD report heightened perceived general interoceptive attention compared to controls, and that perceived general interoceptive attention might differ across different CVD diagnoses. Given content validity issues with the CAQ attention subscale (see “Measuring Interoceptive Attention” section), there has been no research examining cardiac-specific perceived interoceptive attention in individuals with CVD, thus highlighting the need for additional work in this area.

### Takeaways and Future Directions for Research

Existing research suggests that, compared to healthy controls, individuals with CVD report lower actual interoceptive accuracy and higher perceived interoceptive attention. However, despite the growing body of interoception research and the prominence of cardiac-based tasks in measuring interoceptive abilities, there is still a relatively limited body of work examining interoceptive abilities in individuals with CVD. While there is a particular dearth of literature examining perceived interoceptive accuracy and actual interoceptive attention, the field would benefit from additional research on all four core aspects of interoception and further refinement of methods for their measurement. Not only would this contribute to clarifying our understanding of how interoceptive processes outlined in the 2 × 2 factorial model might be altered in the context of CVD, but also it would allow for investigation of discrepancies between the facets [30], which could reveal meaningful clinical implications (see “Clinical Implications and Recommendations” section).

Of the research that does examine interoception in the context of CVD, the approach is split between examining interoceptive abilities in CVD samples that encompass a range of different diagnoses and in samples that have a specific diagnosis that falls under the umbrella of CVD (e.g., heart failure, heart attack, etc.). Future work should consider examining whether there are meaningful differences in interoceptive ability as a function of diagnosis, or whether abilities appear to be consistent across diagnoses within the broader category of CVD. It might also be that depending on the relevant symptomology of specific cardiac diagnoses, certain interoceptive signals (e.g., heartbeat, respiratory stress, dizziness) might be particularly important to monitor from a clinical perspective. If this is the case, development of methods to assess actual accuracy that do not rely on detection of heartbeat might be particularly important for informing clinical work.

Interestingly, despite the high rates of comorbidity between anxiety and CVD, and the established contribution of heart-focused attention to cardiac anxiety [59], there has only been one study to date examining interoception in the context of comorbid CVD and anxiety. Hoffman et al. [53] found that individuals with CHF who were afraid of physical activity demonstrated significantly lower accuracy on a classic HCT than individuals who were not afraid of physical activity. This finding underscores the theoretical relevance of interoceptive processes in the context of comorbid CVD and anxiety symptoms and highlights the need for additional research investigating interoceptive processes in individuals with CVD and anxiety disorders. Given that perceived interoceptive attention has been found to be elevated in both samples with anxiety [44] and CVD [55] compared to controls, this aspect of interoceptive processing might be especially relevant in the context of comorbidity. Specifically, more research is needed to establish (a) whether there is an association between heightened perceived interoceptive attention and anxiety in individuals with CVD, (b) the directionality between heightened interoceptive attention and anxiety in individuals with CVD, and (c) the impact of heightened perceived interoceptive attention on disease management.

## Clinical Implications and Recommendations

Interoceptive processes such as interoceptive accuracy and attention are extremely relevant in the context of disease management, as they impact the way in which the brain senses and makes sense of signals from the body [7]. For example, interoceptive accuracy allows individuals to correctly detect and have confidence in the ability to detect disease-relevant symptomology. Low interoceptive accuracy might therefore result in incorrect detection of the presence or intensity of symptoms and/or a lack of confidence in the ability to notice symptoms. Theoretically, if symptoms are incorrectly detected, decisions about when to seek medical treatment and/or engagement in health-promoting activities (such as physical activity) could be adversely affected. In fact, recent research has shown that low interoceptive accuracy can impair exercise engagement in individuals with CVD, as it has been associated with decreased exercise tolerance in the context of cardiac rehabilitation [52] and increased fear of physical activity [53]. Similarly, attention to interoceptive signals is understood to alter detection and magnitude of sensations on a neurological level [60]. Low attention to interoceptive signals might therefore prevent symptoms from being noticed until they become severe (i.e., intense enough to capture involuntary attention), while hypervigilance to sensations might increase perceived intensity of sensations and result in misclassification of benign sensations as disease-relevant or dangerous [16]. In individuals with CVD, heightened interoceptive attention and lower ability to direct attention away from body sensations have been associated with worse engagement in illness-specific, health promoting behaviors [55], as well as increased stress and symptoms of depression and anxiety [56].

Preliminary research suggests that individuals with CVD might show low interoceptive accuracy and heightened interoceptive attention compared to healthy controls, an interoceptive profile which has been associated with increased risk for health anxiety [48] and which might be maladaptive for promoting the management and recovery of CVD. Heightened interoceptive attention might have a particularly important impact on disease management, though paradoxically, attention to body sensations is necessary for informing disease management (e.g., making diagnoses, changing medications, informing intervention timing; [11, 12]) and also associated with anxiety [48] which worsens cardiac-related outcomes [2, 3]. Given that interoceptive attention appears to have a complex association with disease management, and that individuals with CVD are often asked by medical providers to monitor body sensations as a part of disease management strategy [13], recommendations are needed to promote adaptive interoceptive attention. In order to reduce uncertainty that might lead to hypervigilance to body sensations, which might exacerbate concerns about health, we recommend that providers give patients specific guidelines when recommending symptom monitoring. For example, providers should clearly state (1) which sensations patients should monitor, and how; (2) how often patients should monitor sensations; and (3) for how long after intervention (or change in medication) symptom monitoring is necessary. Where possible, providers should also encourage use of objective methods for monitoring symptoms to account for potential low interoceptive accuracy and/or changes in symptom intensity resulting from hypervigilance to a given sensation [60]. These strategies might encourage a moderate, adaptive level of interoceptive attention and address low interoceptive accuracy, which might help improve disease management and anxiety in the context of CVD.

## Conclusions

In recent years, there has been an increase in research examining interoceptive processes and methods for studying them. While many of the methods utilized rely on cardiac-related interoception (e.g., counting heartbeats, rating confidence in ability to detect heartbeats, examining heartbeat evoked potentials, and assessing attention to cardiopulmonary sensations), our review found that there is a significant lack of research examining interoception in individuals with CVD. Preliminary research on interoception in individuals with CVD has mainly focused on actual interoceptive accuracy and perceived interoceptive attention, and as such more research is needed to understand interoceptive abilities in other domains proposed by the 2 × 2 factorial model and the association between these domains. Currently, there is evidence to suggest that individuals with CVD have low actual interoceptive accuracy and high perceived interoceptive attention when compared to healthy controls. Given that heightened perceived interoceptive accuracy is a well-documented characteristic of health anxiety, medical providers should consider tailoring recommendations for symptom monitoring to increase specificity about which symptoms should be monitored and for how long to promote attention to sensations without hypervigilance. This review and subsequent recommendations represent a first step in applying interoception research to clinical practice for individuals with CVD, while also highlighting the need for continued research within this field.

## Key References

- Hoffman JM, Schulz A, Finke J, Lauterbach M, Schächinger H, Vögele C, Spaderna H. Fear of physical activity relates to cardiac interoception and symptom distress in patients with chronic heart failure. Ment Health Phys Act. 2023; 25:100553. https://doi.org/10.1016/j.mhpa.2023.100553
  - ○ Individuals with high fear of physical activity showed significantly lower actual interoceptive accuracy on the heartbeat counting task than individuals with low fear of physical activity.
- Locatelli G, Ausili D, Lee CS. Association between interoception and self-care in individuals with cardiovascular disease. Eur J Cardiovasc Nurs. 2025; 24:1099-1106. https://doi.org/10.1093/eurjcn/zvaf106
  - ○ Individuals with CVD reported high perceived interoceptive attention and difficulty shifting attention away from body sensations. Difficulty with shifting attention away from body sensations was associated with lower engagement in illness-specific, health-promoting behaviors.
- Trevisan DA, Tsheringla S, McPartland JC. On the relation between interoceptive attention and health anxiety: Distinguishing adaptive and maladaptive bodily awareness. Cogent Psychol. 2023; 10:2262855. https://doi.org/10.1080/23311908.2023.2262855
  - ○ In this cross-sectional study, high interoceptive attention and low interoceptive accuracy were found to predict high health anxiety in a sample of individuals from the general U.S. population.

## Data Availability

No datasets were generated or analysed during the current study.
